# Oxybiotest project: microorganisms under pressure. Hyperbaric oxygen (HBO) and simple pressure interaction on selected bacteria

**DOI:** 10.1186/2045-9912-2-24

**Published:** 2012-09-11

**Authors:** Vincenzo Zanon, Lucia Rossi, Elena Castellani, Enrico Mario Camporesi, Giorgio Palù, Gerardo Bosco

**Affiliations:** 1 ATiP, Diving & Hyperbaric Medicine Center, Padua, Italy; 2 Institute of Microbiology and Virology, Padua Medical School and City Hospital, University of Padova, Padua, Italy; 3 Dept of Biomedical Sciences - Physiological Laboratory, University of Padova, Padua, Italy; 4 CMI, Domus-Medica Diving & Hyperbaric Medicine Unit, Acquaviva, Republic of San Marino; 5 Anesthesiology and Critical Care Medicine, USF Health College of Medicine, Tampa, FL, USA

**Keywords:** Hyperbaric, Oxygen, Microorganism, Bacteria

## Abstract

**Background:**

HyperBaric Oxygen (HBO) therapy involves exposure to pure oxygen in a pressurized room, and it is an already well-established treatment for various conditions, including those originated by serious infections. Starting from the observation of an increased number of patients who were accessing our HBO units for diseases supported from concomitant multidrug-resistant microorganisms, as well as considering the evident clinical benefit and laboratory final outcome of those patients at the end of the treatment, aim of our study was to measure, or better define at least, if there was any interaction between a hyperbaric environment and some selected microorganisms and if those positive results were due to the increased oxygen partial pressure (pO_2_) value or just to the increased pressure, regardless of the fraction of inspired oxygen (FiO_2_) applied (21÷100%).

**Design and methods:**

We applied various increased pO_2_ values in a hyperbaric environment. Our study design was tailored in four steps to answer four specific questions, ordered in a progressive process: OxyBioTest (OBT)-1,2,3, and 4. Specifically, we chose to investigate possible changes in the Minimum Inhibitory Concentration (MIC) and in the Minimum Bactericidal Concentration (MBC) of multi-resistant microorganisms after a single session of hyperbaric therapy.

**Results:**

OBT-1 and OBT-2 provide a semi-quantitative confirmation of the bacterio-cidal and cytostatic effects of HBO. HBO is cidal only if the total exposure pressure is elevated, and cidal or cytostatic effect are not always dependent on the pO_2_ applied.

OBT-4 has shown the adjuvant effect of HBO and antimicrobial drug against some selected bacteria.

**Discussion:**

We seem allowed to hypothesize that only in case of a good approach to a lesion, permitting smaller bacterial loads thanks to surgical debridement and/or eventual antibiotic therapy for example, You can observe the clear effectiveness of the HyperBaric Oxygen (HBO) exposure as a valid adjuvant therapy, even when that lesion is substained from multidrug-resistant micro-organisms. On the contrary when the bacterial load is very high we observe an unchanged situation or a just a slightly diminishing in the number of cfu/ml.

**Conclusions:**

Even if confined in this ‘in vitro’ environment and in a single treatment, just knowing the microorganism strain responsible of the lesion we seem allowed to both weight the possible related effectiveness using HBO Therapy (HBOT) and derive the best pO_2_ to treat the case. A further possible development of the study highlights a comparison between *Acinetobacter baumannii* (ACBA) and *Pseudomonas aeruginosa* (PSAE), and *Escherichia coli* (ESCO) and *Klebsiella pneumoniae* (KLPN).

## Background

We named OxyBioTest (OBT) the present study to better evoke what we were testing: the eventual natural antibiotic capabilities, if any, of either Hyperbaric Oxygen (FiO_2_ 100% under hyperbaric condition) or just pressure itself [normoxic environment (FiO_2_ 21%) under hyperbaric condition].

## Materials and methods

In this study we applied a tailored protocol named OxyBioTest (OBT), investigating potential that hyperbaric oxygen, and/or just pressure itself, could have as natural antibiotic.

The different phases of the study were aimed to evaluate different pressures [1.0, 2.5 and 2.8 ATA; respectively equal to a normobaric environment, and to 15 and 18 meters of water column (msw) or 147.09÷176.50 kPa] and different FiO_2_ (0.21÷1), applied in various combinations upon selected microorganisms. We considered CFU/ml values (colony forming units/ml) and specific stress indexes, as impaired growth or capsule variations.

Upon reviewing the literature [1-6], the aim of the study was to define the application of various increased pO_2_ values, and to determine any interaction between a hyperbaric environment and selected microorganisms. The study design was tailored in four steps to reply to four specific questions, ordered in a progressive process:

OBT-1: a semi-quantitative study of the bacterial replication under hyperbaric conditions after their exposure to 21% and 100% O_2_ (the test has been performed at a 2.5÷2.8 ATA bathymetry and with a manual final plating, during the pure microbiology-lab phase of the test) [7].

OBT-2: same sequence as in OBT-1, but automated final plating to standardize and verify the human variable of the previous procedure [8].

OBT-3: a transition test designed for the experimental material and methods to adopt in the following test (OBT-4), presented as more challenging.

OBT-4: upon positive findings at our first and/or second test, to quantify the adjuvant effect, if any, of HBO and antimicrobic against some selected bacteria.

• OBT-1: In our first experiment we used 5 different microorganism strains [4 American Type Culture Collection (ATCC) strains and 1 multi-sensitive strain isolated from a patient], for 3 three different kinds of bacteria:

a) obligate aerobic (requires oxygen as a source of energy and therefore for growth),

b) anaerobic (derives energy from fermentative processes in the absence of oxygen, it is found in necrotic or abscessed tissues), and

c) facultative anaerobic (derives energy from aerobic or anaerobic metabolism, it includes most of the intestinal pathogens) (Table 1).

**Table 1:** Experiment nr. 1-2: Micro-organisms under hyperbaric test


ATCC 29213	*STaphylococcus AUreus* (STAU)	Gram-positive facultative anaerobic bacteria
ATCC 25922	*EScherichia COli* (ESCO)	Gram-negative facultative anaerobic bacteria
ATCC 27853	*PSeudomonas AEruginosa* (PSAE)	Gram-negative obligate aerobic bacteria
ATCC 29212	*ENterococcus FAecalis* (ENFA)	Gram-positive facultative anaerobic bacteria
BAFR	*BActeroides FRagilis* (BAFR)	Gram-negative anaerobic bacteria

We proceeded as follows [9]:

Step 1: the material

– Selection of the bacterial strains:

ATCC (American Type Culture Collection) 29213 or STAU, ATCC 25922 or ESCO, ATCC 27853 or PSAE, ATCC 29212 or ENFA, and Bacteroides Fragilis (BAFR) from a patient.

– Incubation, at 37°C for 24 hours, of a 2 ml tryptose broth suspensions prepared from these strains and used for the experiment the following day.

– The following day, starting from these bacterial suspensions, we prepared for each group a three step 1:100 serial saline dilution (1998 μl of 0.45% NaCl and 2 μl of bacterial suspension).

Step 2: hyperbaric/normobaric exposure

Four groups of experiments were completed (A_1_, A_2_, B, C):

– A_1_ group: exposure to 100%O_2_ at 2.8 ATA (18 msw; 176.50 kPa) for 75 minutes,

– A_2_ group: exposure to 100%O_2_ at 2.5 ATA (15 msw; 147.09 kPa) for 75 minutes,

– B group: exposure to 21%O_2_ at 2.8 ATA (18 msw; 176.50 kPa) for 75 minutes,

– C group (control): exposure to 21%O_2_ at 1.0 ATA (0 m) for 75 minutes.

In order to limit the bacterial growth, ice packs were applied to the suspensions during their 10 minute transfer (Laboratory/Hyperbaric-Chamber).

Step 3: plating and incubation (60 plates)

– STAU, ESCO, PSAE, ENFA strains

At the end of the exposure to the hyperbaric environment, or to the normobaric conditions, the 3 diluted suspensions for each of the 4 four experimental groups (12 per group, for a total of 48 tubes) were manually streaked on chocolate-agar plates and incubated for 24 hours at 37°C.

– BAFR strain

At the end of the exposure to the hyperbaric environment, or to the normobaric conditions, the 3 diluted suspensions for each of the 4 four experimental groups (12 tubes) were immediately streaked on blood-agar plates and incubated under anaerobic conditions for 24 hours at 37°C.

Step 4: observation of the bacterial growth

• OBT-2: It differs from OBT-1 for the sole step-3, where we verified the weight of the human variable of the procedure via a Walk Away Specimen Processing workstation.

Step 1: see OBT-1. Step 2: see OBT-1.

Step 3: plating and incubation (60 plates)

– STAU, ESCO, PSAE, ENFA strains

At the end of the exposure to the hyperbaric environment or to the normobaric conditions, the 3 diluted suspensions for each of the 4 four experimental groups (12 per group, for a total of 48 tubes) were streaked on chocolate-agar plates and then incubated for 24 hours at 37°C.

– BAFR strain.

At the end of the exposure to the hyperbaric environment or to the normobaric conditions, the 3 diluted suspensions for each of the four experimental groups (12 tubes) were immediately streaked on blood-agar plates and incubated under anaerobic conditions for 24 hours at 37°C.

– In OBT-2, to test the human-operator variable of our previous test, the planting procedure was WASP-assisted, via a Walk Away Specimen Processing workstation that granted the automated pre-analytical plating.

Step 4: see OBT-1.

• OBT-3 : OxyBioTest (OBT)-3 has been used just a warm-up test for the experiment protocol to be used in OBT-4. In OBT-3 we have also tried to evaluate the interaction of oxygen-antibiotic with other methods, such as the Kirby Bauer and the ‘E-Test’.

• OBT-4 : The actual final phase of the study.

Some descriptive notes on the multi-resistant microorganisms we used in this case:

Acinetobacter baumannii (ACBA) - a nonmotile rod, more specifically an obligate aerobic nonfermentative gram-negative pleomorphic bacillus.

Usually found in soil and water, it can survive in soaps and disinfectants. It is found also in humans, and is unknown if it exists as a contaminant or commensal [skin (incidence: up to 25%) and vagina (incidence: 5-15%)]. Acinetobacter family is usually characterized by multi-resistance, especially in strains responsible for nosocomial infections. They can behave as opportunistic pathogens, particularly in burns, debilitated patients, and immunosuppressed patients. It can also cause meningitis, septicemia, endocarditis, soft tissue infections, urinary tract infections and pneumonia.

Klebsiella pneumoniae (KLPN) - capsulated gram-negative facultative anaerobic microorganism. KLPN can be found in the human gastrointestinal tract, skin and nasopharynx. Nevertheless, KLPN may also be found in the environment (water, soil, sewage). The KLPN frequently causes nosocomial infections (urinary tract infections, pneumonia, septicemia, soft tissue infections) which can be fatal in immunocompromised patients.

We chose these two different microorganisms aware that they differ in two fundamental aspects:

ACBA is an obligate aerobic organism while KLPN is a facultative anaerobe;

ACBA is a multi-resistant organism while KLPN acquires mechanisms of drug resistance in relation to the selective pressure of antibiotics administered in hospital. Moreover KLPN is inherently equipped with a capsule whose level of ‘comfort’ or ‘stress’ (in possible future electronic microscope studies) could better depict its progress on the way of a firm multidrug resistance.

Step 1 : Selection of multiresistant bacterial strains from two hospitalized patient samples:

a. Klebsiella pneumoniae (KLPN) identity number 8000938249 (material: perianal swab),

b. Acinetobacter baumannii (ACBA) identity number 8000939446 (material: skin swab).

Step 2: Analysis of the two bacterial strain specific antibiotic sensitivity (Table 2).

**Table 2:** Experiment nr. 4: Antibiotic sensitivity analysis

**Antimicrobial**	**KLPN**		**ACBA**	
	**MIC**	**READING**	**MIC**	**READING**
Amipicillin	≥ 32	resistant	≥ 32	resistant
Amoxicillin/Clavulanic acid	≥ 32	resistant	≥ 32	resistant
Piperacillin	≥128	resistant	≥ 128	resistant
Piperacillin/Tazobactam			≥ 128	resistant
Cefotaxime	16	resistant	≥ 64	resistant
Ceftazidime	≥ 64	resistant	≥ 64	resistant
Cefepime	8	resistant	≥ 64	resistant
Imipenem	≥ 16	resistant	≥ 16	resistant
Meropenem	4	resistant	≥ 16	resistant
Amikacin	8	sensitive	≥ 64	resistant
Gentamicin	≥ 16	resistant	≥ 16	resistant
Levofloxacin	≥ 8	resistant	≥ 8	resistant
Norfloxacin	≥ 16	resistant	≥ 16	resistant
Tigecycline	4	intermediate sensitivity	≤ 0,5	sensitive
Nitrofurantoin	≥512	resistant	≥ 512	resistant
Bactrim	≥320	resistant	≥ 320	resistant
Colistin			1	sensitive

Step 3: Isolation of the two strains in agar-chocolate and subsequent preparation of two bacterial suspensions to be incubated for 24 hours at 37°C.

Step 4: Preparation of 4 MIC (Minimum Inhibitory Concentration) panels for Gram-negative bacilli for each of the two strains (Sensititre® by TREK Diagnostic Systems; see the picture below) and an immediate hyperbaric treatment as follows:

Four groups (A1, A_2_, B, C):

– A_1_ group (2 MICs): exposure to 100%O_2_ at 2.8 ATA (18 msw; 176.50 kPa) for 75 minutes,

– A_2_ group (2 MICs): exposure to 100%O_2_ at 2.5 ATA (15 msw; 147.09 kPa) for 75 minutes,

– B group (2 MICs): exposure to 21%O_2_ at 2.8 ATA (18 msw; 176.50 kPa) for 75 minutes,

– C group (control) (2 MICs): exposure to 21%O_2_ at 1.0 ATA (0 msw) for 75 minutes.

In order to limit the bacterial growth, ice packs were applied to the suspensions during their 10 minute transfer from the Laboratory to the Hyperbaric Chamber.

At the end of the exposure to the hyperbaric environment or to the normobaric conditions, the 8 MIC panels were incubated at 37°C for 24 hours.

Step 5: MIC panel readings followed by agar-chocolate plating of the content of the broth in the microwells where then bacterial growth was inhibited (in order to determine the MBC) and plate incubation for 24 hours at 37°C.

Step 6: MBC (Minimum Bactericidal Concentration) identification.

## Results

– OBT-1 and OBT-2

Showed similar results (Table 3).

OBT-2 confirmed our experimental findings at the end of OBT-1 test, showing results more congruent with the specific characteristic of each group of bacteria.

**Table 3:** Bacteria growth after exposure to Oxygen and/or Pressure


**STAU 29213 - facultative anaerobic**	**Dilution 1**	**Dilution 2**	**Dilution 3**
A_1_ group [100% O_2_ at 2.8 ATA (18 msw; 176.50 kPa)]	10^6 cfu/ml	10^3 cfu/ml	No growth
A_2_ group [100% O_2_ at 2.5 ATA (15 msw; 147.09 kPa)]	10^6 cfu/ml	10^3 cfu/ml	No growth
B group [21% O_2_ at 2.8 ATA (18 msw; 176.50 kPa)]	10^6 cfu/ml	10^3 cfu/ml	No growth
C group [21% O_2_ at 1.0 ATA (0 msw)]	10^6 cfu/ml	10^3 cfu/ml	No growth
**ESCO 25922 - facultative anaerobic**	**Dilution 1**	**Dilution 2**	**Dilution 3**
A_1_ group [100% O_2_ at 2.8 ATA (18 msw; 176.50 kPa)]	10^6 cfu/ml	10^5 cfu/ml	No growth
A_2_ group [100% O_2_ at 2.5 ATA (15 msw; 147.09 kPa)]	10^6 cfu/ml	10^3 ufc/ml	No growth
B group [21% O_2_ at 2.8 ATA (18 msw; 176.50 kPa)]	10^6 cfu/ml	10^5 cfu/ml	10^3 ufc/ml
C group [21% O_2_ at 1.0 ATA (0 msw)]	10^6 cfu/ml	10^3 cfu/ml	No growth
**PSAE 27853 - obligate aerobic**	**Dilution 1**	**Dilution 2**	**Dilution 3**
A_1_ group [100% O_2_ at 2.8 ATA (18 msw; 176.50 kPa)]	10^6 cfu/ml	10^5 cfu/ml	10^4 ufc/ml
A_2_ group [100% O_2_ at 2.5 ATA (15 msw; 147.09 kPa)]	10^6 cfu/ml	10^5 cfu/ml	10^3 ufc/ml
B group [21% O_2_ at 2.8 ATA (18 msw; 176.50 kPa)]	10^6 cfu/ml	10^5 cfu/ml	10^3 ufc/ml
C group [21% O_2_ at 1.0 ATA (0 msw)]	10^6 cfu/ml	10^4 cfu/ml	10^3 ufc/ml
**ENFA 29212 - facultative anaerobic**	**Dilution 1**	**Dilution 2**	**Dilution 3**
A_1_ group [100% O_2_ at 2.8 ATA (18 msw; 176.50 kPa)]	10^6 cfu/ml	10^3 cfu/ml	No growth
A_2_ group [100% O_2_ at 2.5 ATA (15 msw; 147.09 kPa)]	10^6 cfu/ml	10^3 cfu/ml	No growth
B group [21% O_2_ at 2.8 ATA (18 msw; 176.50 kPa)]	10^6 cfu/ml	10^5 cfu/ml	10^3 ufc/ml
C group [21% O_2_ at 1.0 ATA (0 msw)]	10^6 cfu/ml	10^5 cfu/ml	10^3 ufc/ml
**BAFR (from patient) - anaerobic**	**Dilution 1**	**Dilution 2**	**Dilution 3**
A_1_ group [100% O_2_ at 2.8 ATA (18 msw; 176.50 kPa)]	10^4 cfu/ml	No growth	No growth
A_2_ group [100% O_2_ at 2.5 ATA (15 msw; 147.09 kPa)]	10^5 cfu/ml	10^3 cfu/ml	No growth
B group [21% O_2_ at 2.8 ATA (18 msw; 176.50 kPa)]	10^5 cfu/ml	10^3 cfu/ml	No growth
C group [21% O_2_ at 1.0 ATA (0 msw)]	10^6 cfu/ml	10^4 cfu/ml	10^3 ufc/ml

Legend: *cfu* = Colony Forming Unit.

– OBT-3

Confirmed to be just a transition test to perfect the method to apply in the last phase (OBT-4).

The broth suspension in the microwells of our MICs has proved to be more effective as compared to the KB and E-Test. MIC plate allows to simultaneously test multiple antibimicrobial molecules, and is not affected by the human variable (KB-Test requires a lot of experience to run it properly; one thing is to talk about minimal differences between manual and automated WASP-plating procedures in a well-trained team, but quite different to think about the KB-test in the same way).

– OBT-4

**Figure 1:**
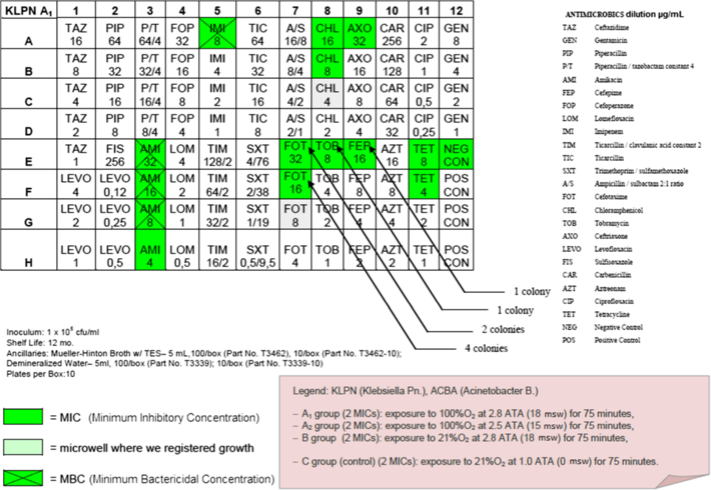
**Klebsiella pneumoniae (KLPN) exposed to 100% O**_**2**_**at 2.8 ATA.**

**Figure 2:**
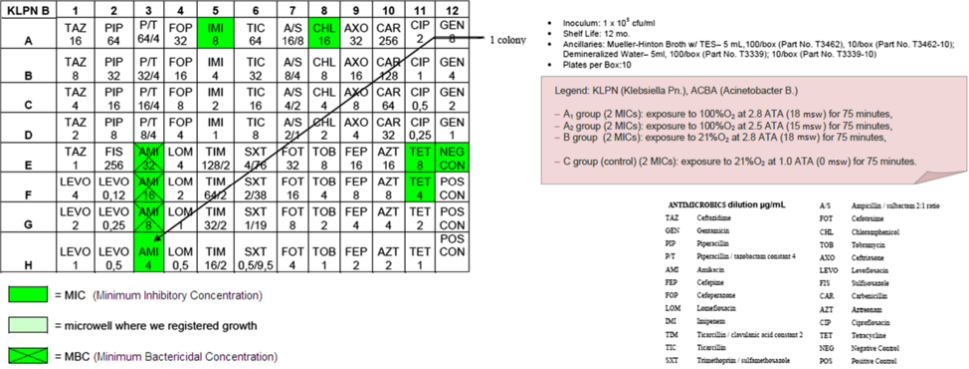
**KLPN plate exposed to 21% O**_**2**_**at 2.8 ATA.**

**Figure 3:**
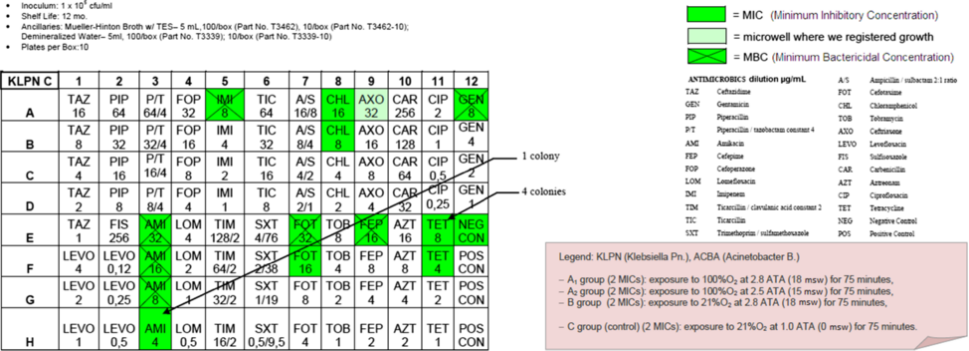
**KLPN plate exposed to 21% O**_**2**_**at 1.0 ATA.**

**Figure 4:**
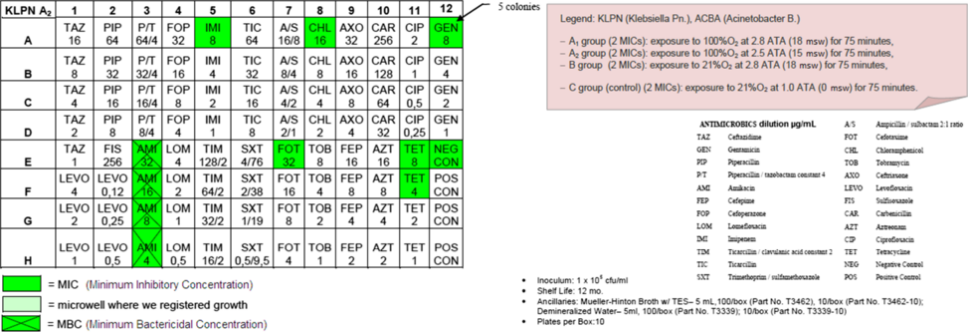
**KLPN plate exposed to 100% O**_**2**_**at 2.5 ATA.**

**Figure 5:**
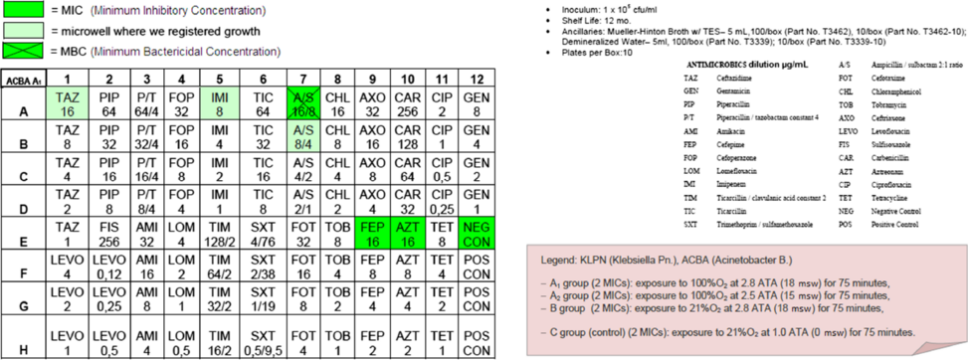
**Acinetobacter baumanii (ACBA) exposed to 100% O**_**2**_**at 2.8 ATA.**

**Figure 6:**
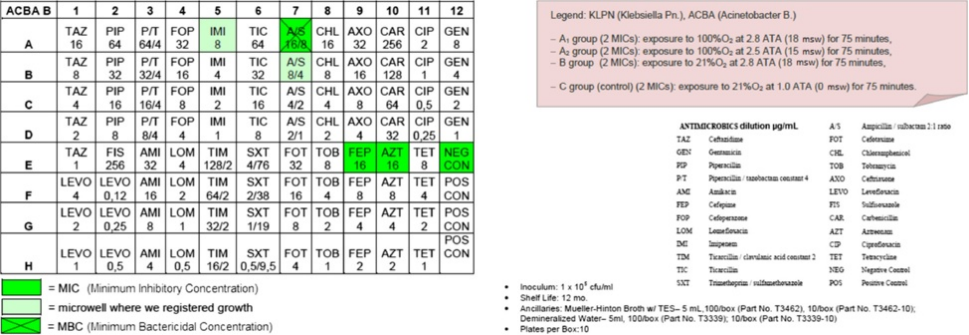
ACBA plate exposed to 21% at 2.8 ATA.

**Figure 7:**
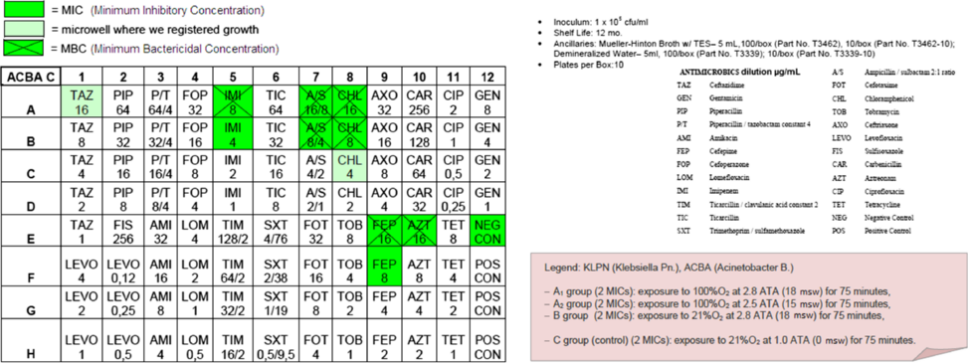
ACBA plate exposed to 21% at 1.0 ATA.

After the semi-quantitative confirmation of the cidal and cytostatic effects of HBO (OBT1 and OBT2), we continued to investigate if there were possible adjuvant interactions between antimicrobial conventional therapy and HBOT. Specifically, we chose to investigate possible changes in the MIC (Minimum Inhibitory Concentration) and in the MBC (Minimum Bactericidal Concentration) of multiresistant micro-organisms after a single session of hyperbaric therapy [Figures 1, 2, 3, 4, 5, 6, 7].

The reason for this interest was the observed increasing number of patients accessing our HBOT Units for other pathologies while infected with concomitant multidrug-resistant microorganisms.

## Discussion

First overall considerations:

– When the bacterial load is very high (as in case in which it is not possible to have a sufficient debridment of the lesion) we observed an unchanged situation or a just a slightly diminishing in the number of cfu/ml.

– As the dilution gets a lower presence of bacteria (2^nd^ and 3^rd^ dilutions; clinically corresponding to a better approach to the lesion) the HyperBaric Oxygen (HBO) exposure can clearly show its effectiveness.

Even if confined in this ‘in vitro’ environment, we seem now allowed, just depending on the microorganism strain responsible of the lesion, to decide if there’s indication or not to use HBO Therapy (HBOT), and which could be the right pO_2_ to apply.

As a direct result of such an exposure to HBO:

– the obligate aerobic microorganism growth (PSAE) is better as pressure and pO_2_ arise

– the facultative anaerobic bacteria, with a common unchanged profile, show different specific behaviors:

STAU > generally speaking, it has no appreciable changes as per growth and cfu/ml,

ESCO > mid dilution shows a longer persistence in higher cfu/ml values at depth, not depending from pO_2_ applied (same at 21% and 100% O_2_ when at 2.8 ATA, while there is a smaller cfu/ml value at 2.5 ATA),

ENFA > 2^nd^ and 3^rd^ dilutions show HBO exposure effectiveness is more dependent on FiO_2_ applied than on the mere pressure increase.

– anaerobic microorganism (as BAFR) gives evidence of an impaired growth and cfu/ml value, apparently directly related to the bathymetry and FiO_2_ applied.

These results indicate that hyperbaric oxygen, generally speaking, is cidal only if the total exposure pressure is elevated.

The automation we used could validate our previous conclusion: the cidal and cytostatic effects of hyperbaric oxygen are more evident as the pressure increases, and are not always depending on which O_2_ tension is applied.

## Conclusions

The first experiment demonstrated that microorganisms exposed to hyperbaric oxygen behave, in relation to bacterial growth, according to their specific physical-biochemical profile.

A cross-check of our results seems lacking in major experimental errors, allowing to conclude HBO is cidal only if the total exposure pressure is elevated, and cidal or cytostatic effect are not always dependent on pO_2_ applied.

The human procedure used, compared to an automated plating system (WASP), showed a not significant margin of error.

Comparing the MIC panels after 24 hours of incubation at 37°C, revealed a different behavior between KLPN and ACBA.

Regarding KLPN, MIC corresponding to the control panel “C” (control group with normobaric oxygen at surface) showed the expected resistance to antibiotics in it, the MIC panel “A_1_” (the compression with 100% O_2_ at 18msw = 176.50kPa) highlighted an attenuation of drug resistance since 4 different antibiotics (AXO, CHL, FOX, TOB) were able to inhibit visible growth of the organism even at concentrations lower than those found in the panel “C” (low MIC). In “A_2_” and “B” panels (100% O_2_ at 15msw = 147.09kPa and 21% O_2_ at 18msw = 176.50kPa) the MICs drug resistances, compared to control, were not reduced while rather slightly wider.

As per ACBA, the MIC corresponding to the control panel “C” (control group with normobaric oxygen at surface) showed the expected resistance to antibiotics in it, the MIC panel “A_1_” (the compression with 100% O_2_ at 18msw = 176.50kPa) highlighted an increase in drug resistance. We registered that 3 different antibiotics (A/S, IMI, EFF) were able to inhibit visible growth of the microorganism only at concentrations greater than those detected in the panel “C” (increase in MIC), and in the case of the antibiotic CHL bacterial growth was visible in all the microwells in the panel. The panel “A_2_” had an increased drug resistance compared with control.

We cannot conclude whether the different behavior of the two microorganisms is due to their different oxygen metabolism (ACBA in this case would increase its resistance as its metabolism is highly dependent on oxygen and ACBA received different but higher pO_2_ exposures than control panel; on the contrary KLPN behavior could probably show to be due to two mechanisms: indirectly stressed not having enough enzyme resources to counteract the O_2_ free radicals, and at the same time KLPN could perceive the more convenient aerobic environment of the chamber as a stress-free situation, lowering its guard (usually expressed as multidrug resistance).

To better study these aspects it would be useful to repeat the experiment introducing other microorganisms to compare to ACBA (obligate aerobic microorganism) and eventually another anaerobic as optional to compare to KLPN. Therefore a possible development could see a comparison between ACBA and PSAE, and eventually ESCO and KLPN (the latter case would be interesting to analyze a multi-sensitive KLPN strain).

## Competing interests

The authors declare that they have no competing interests.

## Authors' contribution

VZ: takes responsibility for the integrity of the work as a whole, from inception to the published article. His authorship credit is based on: the initial study concept and design, drafting the article, and the final approval of the version to be published. Contribution percentage of: 90%. LR: takes responsibility for the integrity of the work as a whole, from inception to the published article. Her authorship credit is based on: supervision of the research group for Microbiology, substantial contributions to the study design, analysis and interpretation of data, revising the article critically for important intellectual content, and the final approval of the version to be published. Contribution percentage of: 90%. EC Authorship credit based on: substantial contributions to the study design, acquisition of data, analysis and interpretation of data, and the final approval of the version to be published. Contribution percentage of: 80%. EMC; Authorship credit based on: supervision of the research group for Hyperbaric, substantial contributions to the study concept and design, analysis and interpretation of data, revising the article critically for important intellectual content, and the final approval of the version to be published. Contribution percentage of: 75%; GP; Authorship credit based on: substantial contributions to the study concept, revising the article critically for important intellectual content, and the final approval of the version to be published. Contribution percentage of: 75%. GB; Authorship credit based on: substantial contributions to the study design, analysis and interpretation of data, and the final approval of the version to be published. Contribution percentage of: 75%.
